# Update on a brain-penetrant cardiac glycoside that can lower cellular prion protein levels in human and guinea pig paradigms

**DOI:** 10.1371/journal.pone.0308821

**Published:** 2024-09-24

**Authors:** Shehab Eid, Wenda Zhao, Declan Williams, Zahra Nasser, Jennifer Griffin, Pavel Nagorny, Gerold Schmitt-Ulms

**Affiliations:** 1 Tanz Centre for Research in Neurodegenerative Diseases, University of Toronto, Toronto, Ontario, Canada; 2 Department of Laboratory Medicine & Pathobiology, University of Toronto, Toronto, Ontario, Canada; 3 Department of Chemistry, University of Michigan, Ann Arbor, Michigan, United States of America; Istituto di Ricerche Farmacologiche Mario Negri IRCCS, ITALY

## Abstract

Lowering the levels of the cellular prion protein (PrP^C^) is widely considered a promising strategy for the treatment of prion diseases. Building on work that established immediate spatial proximity of PrP^C^ and Na^+^, K^+^-ATPases (NKAs) in the brain, we recently showed that PrP^C^ levels can be reduced by targeting NKAs with their natural cardiac glycoside (CG) inhibitors. We then introduced C4’-dehydro-oleandrin as a CG with improved pharmacological properties for this indication, showing that it reduced PrP^C^ levels by 84% in immortalized human cells that had been differentiated to acquire neural or astrocytic characteristics. Here we report that our lead compound caused cell surface PrP^C^ levels to drop also in other human cell models, even when the analyses of whole cell lysates suggested otherwise. Because mice are refractory to CGs, we explored guinea pigs as an alternative rodent model for the preclinical evaluation of C4’-dehydro-oleandrin. We found that guinea pig cell lines, primary cells, and brain slices were responsive to our lead compound, albeit it at 30-fold higher concentrations than human cells. Of potential significance for other PrP^C^ lowering approaches, we observed that cells attempted to compensate for the loss of cell surface PrP^C^ levels by increasing the expression of the prion gene, requiring daily administration of C4’-dehydro-oleandrin for a sustained PrP^C^ lowering effect. Regrettably, when administered systemically *in vivo*, the levels of C4’-dehydro-oleandrin that reached the guinea pig brain remained insufficient for the PrP^C^ lowering effect to manifest. A more suitable preclinical model is still needed to determine if C4’-dehydro-oleandrin can offer a cost-effective complementary strategy for pushing PrP^C^ levels below a threshold required for long-term prion disease survival.

## Introduction

Prion diseases are rapidly progressive and ultimately fatal neurodegenerative diseases observed in several mammalian species. In humans, the lifetime risk to die of these diseases approximates 1 in 5000 [1]. Central to these diseases is the cellular prion protein (PrP^C^), a protein expressed in most cells of the body, with high levels of expression observed in the brain [2,3]. In the disease, PrP^C^ undergoes conformational changes to toxic isomers that have a propensity to form β-sheet-rich assemblies and exert toxicity [4]. Ablation of the prion gene causes no severe phenotypes [5–7] and leads to resistance to prion disease [7–10]. Consequently, any approach that can safely lower PrP^C^ levels represents a potential strategy for the treatment of these diseases.

To date, the most promising strategies reported for lowering PrP^C^ levels silence the expression of the prion gene through customized zinc finger proteins fused to transcriptional repressors [11] or cause the destruction of its mRNAs through optimized antisense oligonucleotides [12]. Although pre-clinical mouse data for these PrP^C^ lowering treatment modalities are promising, how well they will translate to humans remains uncertain. The deployment of genetic approaches is hindered by their less than effective delivery to the human brain, with recombinant adeno-associated virus (rAAV) vector-based gene therapies achieving low transduction efficacies [13] and antisense oligonucleotide (ASO) treatments requiring periodical lumbar punctures [14–16]. Gene therapies are also costly; if existing clinical treatments for spinal muscular atrophy based on these technologies are a guide, the costs per patient may be in the millions of dollars [17]. Critically, even if these challenges can be mitigated, the extent to which PrP^C^ levels need to be lowered before they translate into benefits to patients is uncertain.

A few years ago, we initiated a program to identify a small molecule for lowering PrP^C^ levels. Because repeated attempts to target PrP^C^ with small molecules directly had returned discouraging results [18,19], we considered an indirect targeting strategy. Co-immunoprecipitation work led us to identify Na^+^, K^+^-ATPases (NKAs) in immediate proximity of brain PrP^C^ [20]. This finding spurred the hypothesis that exposure of cells to cardiac glycosides (CGs), the natural inhibitors of NKAs, will lead cells to recycle these protein pumps, with PrP^C^—on account of its spatial proximity to NKAs—being co-internalized and degraded [21]. In the past two years we validated this finding and documented that the cardiac glycoside-mediated degradation of PrP^C^ is largely based on lysosomal degradation, with cathepsin B playing a prominent role in a subset of human cell culture paradigms [21]. Next, we embarked on a search for a CG that exhibits favorable pharmacological characteristics for brain applications. This search culminated in a lead compound with the chemical name C4’-dehydro-oleandrin, which we refer to as KDC203 for brevity. Intriguingly, KDC203 reached higher brain levels, exhibited lower toxicity, and had equal or higher potency for lowering PrP^C^ levels than other CGs [22].

These previous studies had left several questions unanswered: 1) Do CGs lower PrP^C^ levels in a wide range of human cells? 2) How frequently does KDC203 need to be administered for a sustained PrP^C^ lowering effect, and how soon do PrP^C^ levels recover when the treatment is withdrawn? 3) Are guinea pigs a suitable model for evaluating the *in vivo* efficacy of KDC203? Regrettably, mice, the main model for the pre-clinical evaluation of candidate treatments for prion diseases, are refractory to CGs because the most widely expressed α subunit of NKAs, Atp1a1, differs in these rodents by two critical amino acids that govern CG binding in most mammals [23].

The following report provides some answers to these questions. It also revealed that guinea pigs are a better rodent model for studying the effects of CGs on PrP^C^ levels than mice, although they ultimately failed to meet the requirements of a truly effective pre-clinical model for studying the *in vivo* efficacy of KDC203 for the intended application. Encouragingly, we will show that the architecture of the brain and the relatively high NKA levels in this tissue are no hindrance for KDC203 to lower steady-state PrP^C^ levels, so long as effective non-toxic levels of the compound can get there.

## Materials and methods

### Antibodies

*Detection of human and guinea pig PrP*^*C*^: mouse monoclonal anti-PrP (epitope: MKHM), clone 3F4, 1:10000 (catalog number 800310, BioLegend, CA, USA) and mouse monoclonal anti-PrP (epitope: FGSDYEDRYYRE), clone POM1, 1:10,000 (catalog number MABN2285, Millipore Sigma, ON, Canada). A humanized recombinant Fab (epitope: S/NQWNKPS), designated HuM-P, 1:5,000 was generously provided by Joel Watts (University of Toronto, Canada).

*Detection of NKA subunits*: mouse monoclonal anti-ATP1A1, 1:10,000 for cells and 1:20,000 for tissues (catalog number ab7671, Abcam Inc., Toronto, ON, Canada), rabbit polyclonal anti-ATP1A2, 1:10,000 for cells and 1:20,000 for tissues (catalog number ab9094- I, Abcam Inc.), mouse monoclonal anti-ATP1A3, 1:20,000 (catalog number MA3-915, Thermo Fisher Scientific, ON, Canada).

*Neuronal and astrocytic markers*: neurons were detected with the anti-NeuN antibody, 1:10,000 (catalog number EPR12763, Abcam Inc.) or the anti-Tuj-1 antibody, 1:10,000 (catalog number ab78078, Abcam Inc), and astrocytes were detected with the monoclonal anti- GFAP antibody, clone 131–17719, 1:20,000 (catalog number A-21282, Thermo Fisher Scientific).

*Other antibodies*: The autophagy marker LC3A/B was detected in both human and guinea pigs with the rabbit polyclonal anti-LC3A/B, 1:20,000 (catalog number PA116931, Thermo Fisher Scientific). The ER stress marker BiP (GRP78) was detected in both human and guinea pigs with the rabbit polyclonal anti-GRP78, 1:10,000 (catalog number PA1-014A, Thermo Fisher Scientific). The ER stress marker Erp57 was detected in both human and guinea pigs with the mouse monoclonal anti-Erp57, 1:10,000 (catalog number ab13506, Abcam). Secondary antibodies used were goat anti-mouse IgG (H+L)-HRP conjugate (catalog number 1706516, BioRad, Mississauga, ON, Canada) or goat anti-rabbit IgG (H+L)-HRP conjugate (catalog number 1706515, BioRad).

### Cell culture

#### Immortalized cell lines

T98G human glioblastoma cells (catalog number CRL-1690, American Type Culture Collection (ATCC), Manassas, VA, USA), LN-229 glioblastoma cells (catalog number CRL-2611, ATCC), and GPC-16 guinea pig adenocarcinoma cells (CCL-242, ATCC) were cultured in Dulbecco’s Modified Eagle Medium (DMEM) high glucose media (catalog number 11965092, Thermo Fisher Scientific) supplemented with 10% fetal bovine serum (FBS), unless indicated otherwise in the text and figures (catalog number 12483020, Thermo Fisher Scientific), 2 mM glutamax (catalog number 35050061, Thermo Fisher Scientific), and 100 U/mL penicillin & 100 μg/mL streptomycin (catalog number 15070063, Thermo Fisher Scientific). 104C1 guinea pig fetal fibroblast cells (CRL-1405, ATCC) were cultured in Roswell Park Memorial Institute (RPMI)-1640 media (catalog number 11875093, Thermo Fisher Scientific), supplemented with 10% FBS, 2 mM glutamax, and 100 U/mL penicillin & 100 μg/mL streptomycin. Cells were maintained at 37°C and 5% CO2 in a humidified incubator and were passaged at 1:10 dilutions every 3–4 days using 0.25% trypsin-ethylenediamine tetraacetic acid (EDTA) (catalog number 25200056, Thermo Fisher Scientific).

#### Primary cortical neurons

Guinea pig primary cortical neuron cultures were established from P40 embryos. One day before harvesting primary neurons, 60 mm Nunc plates (catalog number 150462, Thermo Fisher Scientific) were coated overnight at room temperature with 3 mL of 50 μg/mL poly-D-lysine hydrobromide (PDL) (catalog number P6407, Sigma-Aldrich, Oakville, ON, Canada) that was diluted in phosphate buffered saline (PBS) (catalog number D8537-500 ML, Sigma-Aldrich). The next day, PDL-coated plates were washed 4x with sterile distilled H_2_O (catalog number 15230162, Thermo Fisher Scientific) and left to dry in a biosafety cabinet. To harvest neurons, a pregnant Hartley guinea pig was deeply anesthetized using isoflurane (catalog number CP0406V2, Fresenius Kabi, Bad Homburg, Germany). Next, the embryos were harvested and placed in ice-cold DMEM. Embryos were transferred to a sterile biosafety cabinet where brains were rapidly extracted and placed in ice-cold DMEM. Under a Leica MS5 stereo Microscope (MS5-PS, Leica Microsystems, Morrisville, USA) the cerebellum and meninges were removed. Cortices were transferred to a 15 mL conical tube and enzymatically digested using a 0.22 μm filtered solution of 0.01% (w/v) papain (catalog number P5306, Sigma-Aldrich), 0.1% (w/v), Neutral Protease (catalog number P4630, Sigma-Aldrich), 0.01% (w/v), and DNase (catalog number DN25, Sigma-Aldrich) for 25 minutes at 37°C. Next, cortices were triturated using a 5 mL pipette, then a P1000 pipette, and finally a glass-fired Pasteur pipette. After a brief centrifugation at 300 rcf for 5 minutes, fresh DMEM, supplemented with 50% FBS (catalog number 12483020, Thermo Fisher Scientific), was added to each conical tube. Cells were plated at a density of 5x10^5^ per cm^2^. Two hours after plating, the media were replaced with fresh neurobasal medium (catalog number 21103049, Thermo Fisher Scientific) supplemented with 1x B27 (catalog number 17504044, Thermo Fisher Scientific), 2 mM glutamax, and 100 U/mL penicillin & 100 μg/mL streptomycin. 1 μM cytosine β-D-arabinofuranoside (AraC) (catalog number C1768, Sigma Aldrich) was added to neuronal cultures 3 days after plating to limit glial proliferation.

#### Primary cortical astrocytes

Guinea pig primary cortical astrocyte cultures were also established from P40 embryos and were harvested by mechanical digestion. To this end, the brains were initially cut into smaller sections and subsequently finely minced using a scalpel, before being transferred to a 15 mL conical tube with DMEM. Next, the digested material was passed through a 70 μm cell strainer and collected in a fresh 15 mL conical tube. After a brief centrifugation at 300 rcf for 5 minutes, the medium was replaced with fresh DMEM, supplemented with 10% fetal bovine serum, 2 mM glutamax, and 100 U/mL penicillin & 100 μg/mL. Cells were plated and media were replaced one week later, at which point the plates were confluent.

#### Primary cardiomyocytes

Primary cardiomyocytes were cultured according to an established protocol [24]. One day prior to harvest, 60 mm Nunc plates (catalog number 150462, Thermo Fisher Scientific) were coated with 5 mL of autoclaved 1% gelatin (catalog number G2500, Sigma-Aldrich) and placed for 2 hours at 37°C. Next, the gelatin solution was removed, and plates were stored at room temperature until use the next day. Embryonic guinea pig hearts were extracted and minced into 1 mm^2^ pieces in ice-cold DMEM using scalpels. Next, enzymatic digestion was conducted using 0.5 mg/mL collagenase Type II (catalog number COL005, BioShop Burlington, ON, Canada) diluted in Hanks Balanced Salt Solution (HBSS) (catalog number 14025092, Thermo Fisher Scientific) for 10 minutes at 37°C while shaking at 100 rpm. Next, the material was triturated using a fire-blown glass Pasteur pipette and larger material was allowed to settle. The supernatant was transferred to a 15 mL conical tube (catalog number 62.554.205, Sarstedt Inc., Montreal, QQ, Canada). Collagenase solution was twice re-added as before to the larger tissue to harvest cardiomyocytes efficiently. The single-cell suspension was briefly centrifuged for 5 minutes at 300 rcf and the supernatant (containing mostly fibroblasts and endothelial cells) was discarded. The remaining cell pellet (containing mostly cardiomyocytes) was resuspended in 20 mL of plating medium, composed of DMEM supplemented with 10% FBS. To separate the cardiomyocytes from any remaining contaminant fibroblast cells, a pre-plating method was employed. This method leverages the fact that fibroblasts adhere to uncoated cell culture plates, while cardiomyocytes only attach to coated plates. The cells were plated onto two 100 mm uncoated plates for 2 hours at 37°C to allow fibroblasts to attach to the plate. After 2 hours, the media (containing a cardiomyocyte suspension) was isolated, and the pre-plating step was repeated. The remaining cardiomyocytes were then counted using countess (catalog number, Thermo Fisher Scientific) and plated at 2.2 million cells per 60 mm Nunc plate coated with 1% gelatin.

### Cell treatments

#### CG treatments

One day before treatment with KDC203 or Ouabain, maintenance media for each cell type were replaced with serum-free media or media supplemented with 1%, 2%, or 10% horse serum (catalog number 26050088, Thermo Fisher Scientific) or fetal bovine serum, as indicated in the text or figures. For primary neuronal cultures, media conditions were not changed. The following day, CGs were introduced into pre-warmed media at specified concentrations within a conical tube, maintaining the dimethylsulfoxide (DMSO) (catalog number DMS555.250, BioShop) concentration at 0.1%. After agitating the conical tubes for 10 seconds with a vortex to achieve a homogeneous CG solution, the mixture was added to the cells. Treatment continued for one to 7 days, as indicated in the text and figures, during which 50% of the media containing the appropriate CG concentration was replenished every 2 days. Vehicle-treated cells and organotypic brain slices were subject to the same DMSO concentration (0.1%) in their media.

#### PI-PLC treatment

For the enzymatic treatment with phosphoinositide-specific phospholipase-C (PI-PLC)(catalog number P6466, Thermo Fisher Scientific), cells were first cultured and treated as described above. At the end of these treatments, media were aspirated, and cells were washed twice with PBS. Next, 0.25 U of PI-PLC in 1 mL of PBS were added to each 60 mm cell culture plate, after which the cells were placed on a rocker for 30 minutes in a 4°C cold room. Next, plates were gently tilted, and the supernatant was collected and transferred to a 5 mL Eppendorf tube (catalog number 0030119401, Eppendorf, Mississauga, ON, Canada). Cells were then washed twice with PBS and lysed as described below. To each 1 mL of supernatant (containing cell surface PrP^C^ that was released by PI-PLC), 4mL of pre-chilled acetone (catalog number A18P4, Thermo Fisher Scientific) were added, gently vortexed, and left overnight at -20°C. The following day, samples were centrifuged for 30 minutes at 7000 rcf. The supernatants were decanted, and the remaining pellets were first air-dried, then solubilized in 50 μL of 1× Bolt LDS buffer (catalog number B0007, Thermo Fisher Scientific). Finally, samples were transferred to a 1.5mL microcentrifuge tube (catalog number 1210–001, FroggaBio, Concord, ON, Canada), heated at 70°C for 10 minutes, then analyzed by sodium dodecyl-sulfate polyacrylamide gel electrophoresis (SDS-PAGE) and western blotting.

#### PNGase F treatment

To remove N-glycans from PrP^C^, enzymatic digestions were undertaken with Peptide: N-glycosidase F (PNGase F) according to the manufacturer’s protocol (catalog number P0704S, New England Biolabs, Whitby, ON, Canada) with minor modifications. Briefly, equal amounts of protein, i.e., 40 μg, were denatured using 10× Denaturing Buffer at 95°C for 10 minutes. Next, 10× GlycoBuffer 2 and 10× NP40 were added. Samples were briefly vortexed, then 0.5 μL of PNGase F enzyme was added. Samples were incubated for at least 2 hours at 37°C while shaking in a ThermoMixer F (catalog number 5384000020, Eppendorf) at 600 rpm. Next, samples were briefly centrifuged and an appropriate amount of 4× Bolt LDS buffer (catalog number B0007, Thermo Fisher Scientific) was added to achieve a final concentration of 1× LDS, before proteins were analyzed by SDS-PAGE and western blotting.

#### Endo H treatment

Endo H treatment was conducted according to manufacturer’s protocol (catalog number P0702, New England Biolabs). Briefly, equal amounts of protein, i.e., 40 μg, were denatured using 10× Denaturing Buffer at 95°C for 10 minutes. Next, 10× GlycoBuffer 3 was added. Samples were briefly vortexed, then 1 μL of Endo H enzyme was added. Samples were incubated for at least 2 hours at 37°C while shaking in a ThermoMixer F at 600 rpm. Next, samples were briefly centrifuged and an appropriate amount of 4× Bolt LDS buffer was added to achieve a final concentration of 1× LDS, before proteins were analyzed by SDS-PAGE and western blotting.

### Brain slice cultures

Organotypic cerebellar and cortical slices were harvested from postnatal Day 0 to Day 2 Hartley guinea pigs pups using a procedure built on a previously published protocol [25]. Specifically, guinea pig pups were deeply anesthetized using 5% isoflurane. Brains were then harvested and immersed in an ice slurry of HBSS (catalog number 14065056, Thermo Fisher Scientific) which was supplemented with 25 mM sodium bicarbonate (catalog number S5761, Sigma-Aldrich), 33 mM glucose (catalog number A2494001, Thermo Fisher Scientific) and 1mM kynurenic acid (catalog number K3375, Sigma-Aldrich) and carbonated for 30 minutes using 5% CO2 (catalog number 100906, Messer Canada, Etobicoke, ON, Canada) and 95% oxygen. The brain was mounted onto a Leica specimen disc and 300 μm thick cerebellar or 250 μm thick cortical slices were cut with a vibratome (catalog number VT1000, Leica, Richmond Hill, ON, Canada) while submerged in the dissection solution. Individual slices were transferred to a 6-well, 30 mm, PTFE, 0.4 μM Millicell-CM membrane insert (catalog number PICM0RG50, Sigma-Aldrich). Any residual dissection solution was removed using a disposable Pasteur pipette, and the inserts were transferred to a 6-well cell culture plate (catalog number 83.3920.500, Sarstedt, Montreal, QC, Canada) containing 1.1 mL of slice culture medium composed of 50% (v/v) minimal essential medium (MEM) (catalog number 11095080, Thermo Fisher Scientific), 25% (v/v) DMEM and 25% (v/v) horse serum (catalog number 26050088, Thermo Fisher Scientific) supplemented with 0.65% (w/v) glucose), 2 mM glutamax, and 100 U/mL penicillin & 100 μg/mL streptomycin. Slices were maintained at 37°C and 5% CO2 in a humidified incubator. Cell culture media were refreshed every 2 days. Cardiac glycoside treatments began 14 days after harvesting of the slices and continued for 2 weeks with fresh media and their CG content being replaced every 2 days. For protein analysis, organotypic slices were rinsed twice with ice-cold PBS, then scraped into a 1.5 mL microcentrifuge tube. Following a brief spin, PBS was aspirated and replaced with 100 μL ice-cold lysis buffer containing 0.5% Nonidet P-40 (NP40), 0.5% deoxycholate (DOC), 150 mM NaCl, 50 mM Tris / HCL, pH 8.3, supplemented with protease inhibitor cocktail (catalog number 11836170001, Sigma-Aldrich). To facilitate cell lysis, slices were subjected to 3× freeze-thaw cycles and 3× 30 seconds of sonication. Samples were placed on ice for 30 minutes with 15 seconds agitation by vortexing every 10 minutes. Insoluble cellular debris was removed through 5-minute centrifugation at 2,000 rcf, followed by 15-minute centrifugation at 16,000 rcf. Protein concentrations of the supernatants were determined by the bicinchoninic acid assay (BCA) using the Pierce BCA Protein Assay Kit (catalog number 23225, Thermo Fisher Scientific).

### Animals

Dunkin-Hartley guinea pigs (GPs) were obtained from Charles River Laboratories and housed two per cage on a 12-hour artificial day/night cycle. Cages were bedded with sawdust and were changed once a week. GPs received daily health checks from Animal Resource Centre staff. Drinking water and protein chow (18%) was available *ad libitum*. All animal procedures were in accordance with the Canadian Council on Animal Care, reviewed and authorized by the University Health Network Animal Care Committee and approved under Animal Use Protocol 6491.

Alzet osmotic pumps (catalog number 2ML1, Durect Corporation, Cupertino, CA, USA) with a volume of 2 ml were filled with KDC203 solubilized in a 1:1 ratio of sterile DMSO and polyethylene glycol 400 (PEG400) (catalog number PHR2891, Sigma-Aldrich). In accordance with manufacturer guidelines, pumps were filled and then submerged in 0.9% NaCl (saline) and incubated at 37°C one day prior to surgical implantation. On the day of surgery, guinea pigs were deeply anesthetized after placement in a chamber with 5% Isoflurane. Next, a small patch of hair on the dorsal side of the guinea pigs was shaved, the guinea pigs were placed on a heating pad, and anesthesia was maintained at 2% Isoflurane levels using a nosecone. Finally, 0.3 mg/kg of Meloxicam (catalog number 02240463, Boehringer Ingelheim) was administered subcutaneously, and Optixcare lubricant (catalog number 876198042524, CLCMEDICA) was applied to the eyes. The surgical incision site was sterilized using povidone-iodine (catalog number 109–08, SCIsupply Inc., Barrie, ON, Canada) and 70% Isopropyl Alcohol (catalog number P010IP70, Commercial Alcohols Inc., Toronto, ON, Canada). Next, a scalpel was used to make a 1 cm incision and large scissors were used to create a pocket in the subcutaneous region that could accommodate the Alzet pump implant for drug release into the subcutaneous cavity. After implantation, a surgical wound clip applier (catalog number RS-9260, Fisher Thermo Scientific) and 9 mm wound clips (catalog number RS9262, Fisher Thermo Scientific) were used to close the incision site. Topical bupivacaine was applied around the incision site and 5 mL of 0.9% saline was administered subcutaneously. Guinea pigs remained on the heating pad until ambulatory, at which point they were transferred to housing cages. Ambulatory guinea pigs were monitored hourly until complete recovery from anesthesia. Oral meloxicam was administered daily for 2 days after surgery. Control guinea pigs were implanted with an osmotic pump filled with formulation only, i.e., 1:1: DMSO and PEG400 (v/v). After 7 days of treatment, guinea pigs were deeply anesthetized and underwent transcardiac perfusion with PBS. The brain, heart, lungs, kidney, and liver were harvested and immediately snap-frozen in liquid nitrogen until further processing.

### Western blotting

Cells were washed twice using ice-cold PBS and lysed in ice-cold lysis buffer containing 0.5% (w/v) NP-40, 0.5% (w/v) DOC, 150 mM NaCl, 50 mM Tris/HCL, pH 8.3, supplemented with protease inhibitor cocktail (catalog number 11836170001, Sigma-Aldrich). Following 30-minute incubation on ice, with agitation by 15 second vortexing every 10 minutes, cellular debris was removed through a 5-minute spin at 2000 rcf, followed by a 10-minute spin at 16,000 rcf. After removal of the supernatant, protein concentration was adjusted using lysis buffer, determined by the bicinchoninic acid assay (BCA) employing the Pierce BCA Protein Assay Kit (catalog number 23225, Thermo Fisher Scientific). Subsequently, an equivalent volume of lysate was incubated with 2× Bolt LDS buffer (catalog number B0007, Thermo Fisher Scientific) and heated for 10 minutes at 70°C. The final protein concentration in LDS buffer was maintained at 1–2 μg/μL. Guinea pig tissue was homogenized in 7 mL Tissue Homogenizing Tubes (catalog number P000935-LYSK0-A, Bertin Technologies, Rockville, MD, USA) containing ice-cold 150 mM NaCl, 50mM Tris/HCl, pH 8.3, and supplemented with a protease inhibitor to 20% weight/volume (w/v). Homogenization was conducted using a Minilys homogenizer (catalog number P000673-MLYS0-A, Bertin Technologies) with three sets of 1-minute bead-beating pulses. Next, an equal volume of 2× lysis buffer (1% NP40, 1% DOC, 150 mM NaCl, 50 mM Tris/HCL, pH 8.3) was added to the 20% homogenate. Lysates were incubated on ice for 30 minutes, then insoluble debris was removed through a 5-minute spin at 2000 rcf, followed by a 10-minute spin at 16,000 rcf. Downstream analyses were identical to those of cell lysates described above.

For analysis of cell and tissue lysates, proteins were separated by sodium dodecyl-sulfate polyacrylamide gel electrophoresis (SDS-PAGE) on 10% or 4–12% Bolt Bis-Tris gels (catalog number NW00105BOX, Thermo Fisher Scientific) using 1x MES SDS running buffer (catalog number NP0002, Thermo Fisher Scientific). The SDS-PAGE run was performed using a Mini Gel Tank (catalog number A25977, Thermo Fisher Scientific) at 165 volts for 40 minutes. Next, the gels were briefly rinsed in distilled water and proteins were transferred to 0.45 μm polyvinylidene fluoride (PVDF) membranes (catalog number IPVH00010, MilliporeSigma, Burlington, ON, Canada) using Tris-Glycine transfer buffer (25 mM Tris, 192 mM glycine) with 20% methanol. The transfer was conducted for 1 hour at 35 V. Next, the PVDF membrane was briefly rinsed with distilled water, then blocked for 1 hour in 5% skimmed milk diluted in Tris-buffered saline (25mM Tris, 150mM NaCl) with 0.1% Tween-20 (TBST) (catalog number TWN508, BioShop). Membranes were then incubated with primary antibodies diluted in 5% skimmed milk and left overnight at 4°C with gentle rocking. The next day, membranes were washed four times in TBS, then incubated with the corresponding horseradish peroxidase (HRP)- conjugated secondary antibodies in 5% skimmed milk for 1 hour at room temperature. Subsequently, membranes were washed four times with TBST, briefly pat dried for 5 seconds using KimWipes (catalog number 34155, Kimberly-Clark Professional, Mississauga, ON, Canada), and incubated for 1 minute with 1 mL of enhanced chemiluminescence (ECL Pro) reagent (catalog number NEL122001EA, PerkinElmer Health Sciences Canada Inc., Woodbridge, ON, Canada). Finally, membranes were exposed to autoradiography film (catalog number MED-CLMS810, Mandel Scientific, Toronto, ON, Canada) and developed using a film developer (Model SRX-101A, Konica-Minolta).

### Statistical analyses

Comparisons of steady-state protein levels by western blot analyses made use of a minimum of three biological replicates. Densitometry analyses of western blot signals were undertaken with ImageJ software (version 1.53e). Each treatment replicate was compared to a control replicate that was run on the same gel. In assessing various concentrations of KDC203, we assumed normal Gaussian distribution and equal variance. A one-way ANOVA was applied to examine the null hypothesis. When rejected, an unpaired two-tailed *t*-test was used for pairwise comparisons to determine *p*-values. All statistical analyses were performed using GraphPad Prism software (version 10.1) with a *p* < 0.05 significance threshold. One (*), two (**), three (***), and four (****) asterisks signify *p*-values of <0.05, <0.005, <0.0005, and <0.00005, respectively. The abbreviation ‘ns’ is shown when *p*-values did not meet the significance threshold.

## Results

### The PrP^C^ lowering potency of KDC203 affects all post-translational PrP^C^ isoforms, is reduced in rich culture media, and may only be apparent when analyzing the cell surface pool of PrP^C^

**Fig 1:**
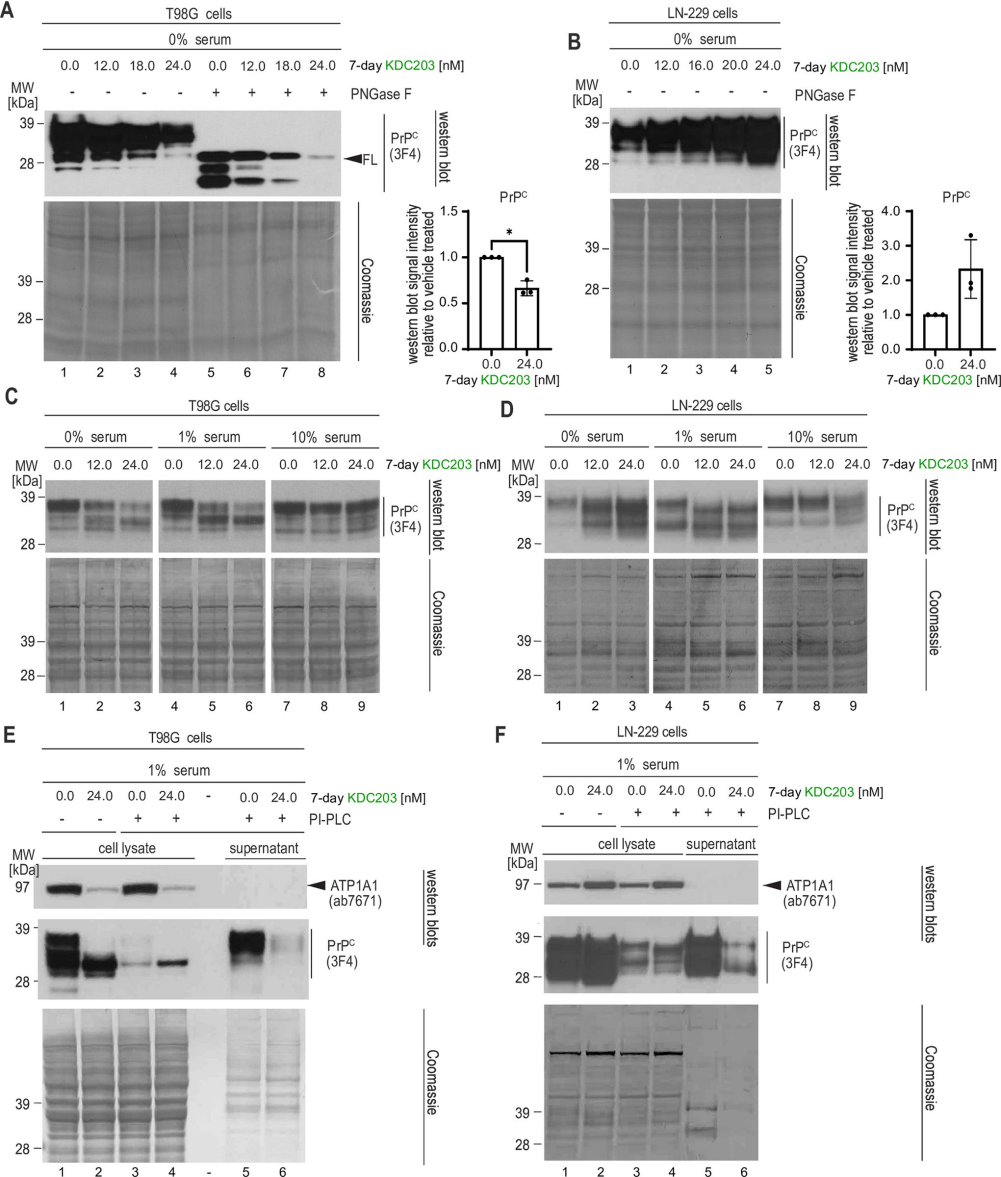
The PrP^C^ lowering potency of KDC203 affects all post-translational PrP^C^ isoforms but is reduced in rich culture media. (A) KDC203 concentration-dependent reduction of all PrP^C^ isoforms is apparent in PNGase F treated human T98G cell extracts. The Coomassie stain documents equal loading. (B) Human LN-229 cells exhibit a KDC203 concentration-dependent increase in total PrP^C^ levels. (C) The capacity of KDC203 to lower PrP^C^ levels in T98G cells is reduced in serum-rich media. (D) The KDC203-dependent increase of PrP^C^ levels in LN-229 levels is reduced in serum-rich media. (E) The cell surface pool of PrP^C^ that can be released into the supernatant by PI-PLC digestion dominates total PrP^C^ levels in T98G cells and is strongly reduced upon KDC203 treatment. ATP1A1 levels are also reduced in KDC203-treated T98G cells but cannot be observed in the PI-PLC digestion supernatant. (F) KDC203 treatment caused an increase in total ATP1A1 and PrP^C^ levels in LN-229 cells yet reduced the PI-PLC-releasable cell surface pool of PrP^C^.

To learn how PrP^C^ levels respond to KDC203 treatment in a broader selection of human cells, we treated eight widely used human cell models (ReN VM, T98G, SH-SY5Y, HeLa, HEK293, U2OS, LN-229, BE(2)-M17) with KDC203 and observed two types of responses. In six cell models, i.e., all models except for LN-229 and U2OS cells, KDC203 exposure led to a dose-dependent reduction in steady-state PrP^C^ levels. T98G cells are representative of a type of response seen in many human cell models (**Fig 1A**), which we had previously reported in ReN VM cells [22]. PNGase F digestion of T98G cell extracts revealed that 7-day KDC203 exposure diminishes not only the levels of full-length (FL) PrP^C^ but also its C-terminal fragments. In contrast, LN-229 cells and U2OS cells (not shown) responded with a KDC203 dose-dependent increase in steady-state PrP^C^ levels (**Fig 1B**). To investigate if serum content of the media can influence this divergence, we next switched T98G or LN-229 cells from maintenance medium containing 10% serum to media supplemented with 0%, 1%, or 10% serum, then treated them with KDC203 or Ouabain the next day.

In T98G cells, as serum levels increased, KDC203 was observed to be less potent in its dose-dependent lowering of total PrP^C^ levels but no reversal of the effect was observed. For instance, at 1% serum levels, the slowest migrating PrP^C^-antibody reactive band was still diminished with increases in KDC203, however, the fast migrating band became more pronounced than the corresponding band seen at 0% serum levels (**Fig 1C, compare lanes 2 and 3 with lanes 5 and 6**). This trend became most striking at 10% serum levels, which essentially blocked the KDC203-dependent lowering of steady-state PrP^C^ levels. In contrast, when LN-229 cells were grown in 0% serum, we again observed the accumulation of steady-state PrP^C^ levels (**Fig 1D, lanes 1–3**). This accumulation was lower in LN-229 cells grown in media supplemented with 1% serum and was all but lost in the presence of 10% serum. These experiments indicated that increased serum levels render KDC203 less potent in both cell lines but could not explain why KDC203 causes PrP^C^ lowering in T98G cells whereas in LN-229 cells the opposite outcome is observed.

Next, we hypothesized that these different outcomes may reflect differences in the respective reactions of the two cell types to the KDC203-induced removal of PrP^C^ from the cell surface, rather than the cell surface removal itself. Specifically, we considered that the absence of full-length PrP^C^ bands in the LN-229 cells may indicate that cell surface PrP^C^ has been removed also in this cell model and that the faster migrating bands may represent intracellular PrP^C^. To address this point experimentally, we added a phosphoinositol phospholipase C (PI-PLC) cleavage step to the workflow prior to the cell harvest step, then analyzed cell lysates and PI-PLC releasable supernatants by western blotting. This experiment recapitulated the KDC203-induced drop versus increase in total PrP^C^ levels in T98G cells and LN-229 cells, respectively (**Fig 1E and 1F, lanes 1 and 2**). These trends were not limited to PrP^C^ but extended to ATP1A1, the main NKA α-subunits targeted by CGs. Critically, the side-by-side examination of the PI-PLC releasable pool of PrP^C^ established that cell surface levels were greatly diminished in both T98G and LN-229 cells when cells were treated with KDC203 (**Fig 1E and 1F, lanes 5 and 6**). Consequently, the accumulation of total PrP^C^ signal observed in LN-229 cells reflected a shift in the balance between the cell surface replacement and degradation of internalized PrP^C^ in KDC203-treated cells that caused a net increase in intracellular PrP^C^, as opposed to an inability of KDC203 to remove PrP^C^ from the cell surface in this cell model.

### KDC203 treatment lowers PrP^C^ levels in guinea pig cells and PrP^C^ expression temporarily overshoots when KDC203 is withdrawn

**Fig 2:**
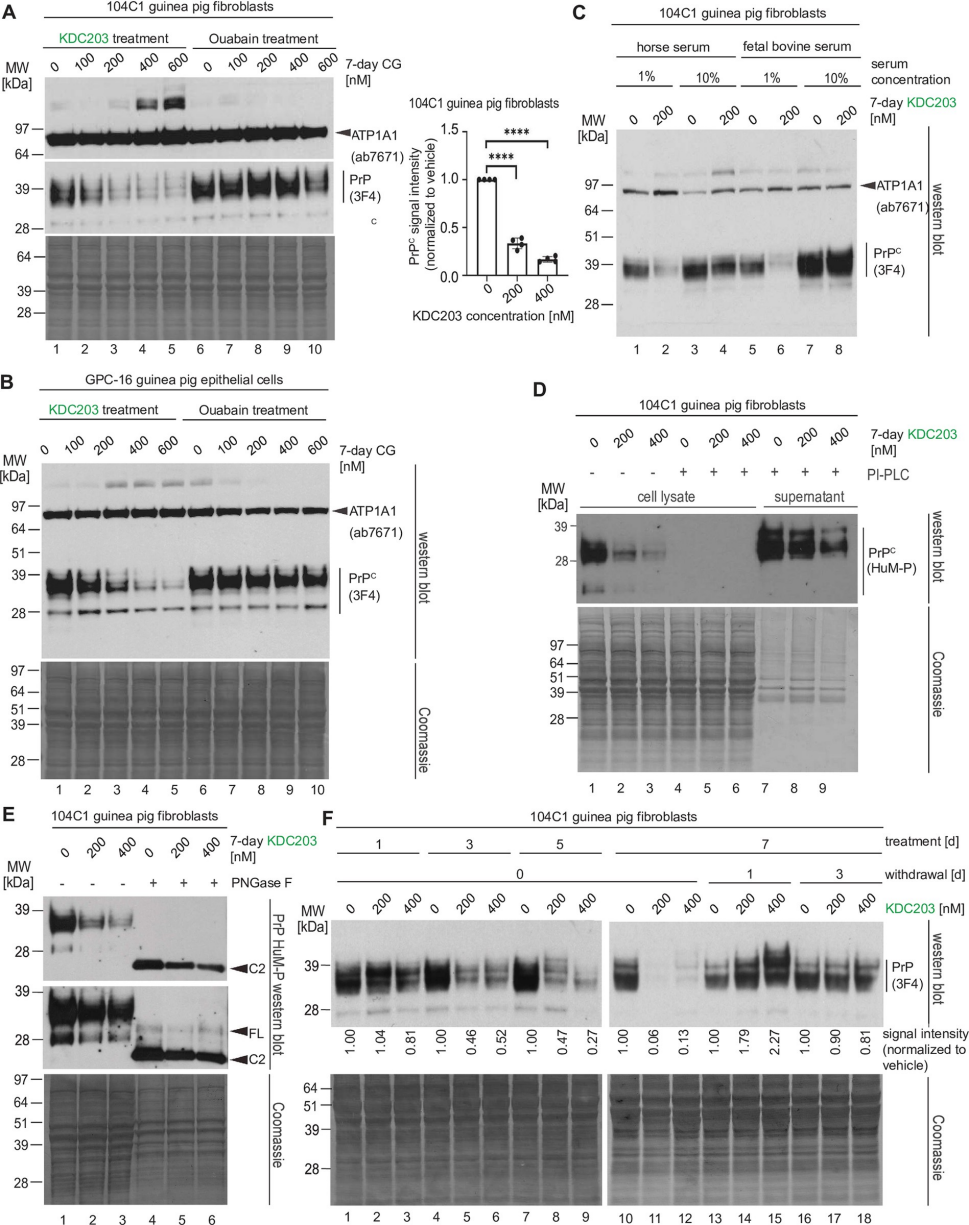
KDC203 treatment lowers PrP^C^ levels in guinea pig cells and PrP^C^ expression temporarily overshoots when KDC203 is withdrawn. (A) The guinea pig cell line 104C1 which exhibits a fibroblast morphology responds to 7-day treatment with KDC203, but not Ouabain, with a significant reduction in its PrP^C^ levels. However, the KDC203 concentrations required for this outcome exceed approximately 30-fold those observed to achieve a similar PrP^C^ reduction in human cells. The asterisk indicates high molecular weight ATP1A1 antibody-reactive bands in a subset of fractions. Although the identity of these bands has not been resolved, their presence is consistent with partially SDS-stable ATP1A1-containing complexes. (B) The epithelial guinea pig cell line, GPC-16, resembles 104C1 cells in their response to KDC203 or Ouabain. A Coomassie stain of the western blot membrane validated equal protein loading. ATP1A1 signals were revealed following a reprobe of the PrP^C^ blot. (C) Guinea pig 104C1 cells recapitulate reduced PrP^C^ lowering potency of KDC203 when grown in media with higher serum levels. Note that the relatively stable ATP1A1 levels serve partially as a loading control as they were revealed on the same membrane following a reprobe of the PrP^C^ blot. (D) Upon PI-PLC cleavage of guinea pig 104C1 cells, no PrP^C^ can be detected in cellular lysates, and the PI-PLC releasable pool is diminished in KDC203 treated cells. (E) By its apparent molecular weight, PrP^C^ expressed in guinea pig 104C1 cells is predominantly composed of the N-terminally truncated C2 isoform. (F) Daily KDC203 treatment of guinea pig 104C1 cells caused a time-dependent reduction of steady-state PrP^C^ levels, culminating in a profound reduction after 7 days of treatment. The subsequent withdrawal of KDC203 reveals a temporary upregulation of the underlying PrP^C^ expression whose magnitude correlates with the prior KDC203 concentration used to achieve the PrP^C^ lowering effect. This effect ceases after 3 days of KDC203 withdrawal.

Guinea pigs are known to be more responsive to CGs than mice and to be infectable with prions. As such, guinea pigs possess two characteristics that may make them a suitable pre-clinical model for testing if KDC203 treatment can lower PrP^C^ levels and extend prion disease survival. As a precursor to working with guinea pigs, we obtained the only two guinea pig-derived cell models available through ATCC and repeated some of the work we had already undertaken with human cells. When exposing 104C1 cells that acquire a fibroblast morphology to increasing concentrations of KDC203, we observed highly significant (*p*<0.00005) dose-dependent reductions in PrP^C^ levels (**Fig 2A**). However, the KDC203 doses required to achieve this outcome were approximately 30-fold higher than the concentrations required for achieving similar steady-state PrP^C^ level reductions in human cells. The parallel treatment of 104C1 cells with identical concentrations of Ouabain did not lower PrP^C^ levels. Analogous results were obtained with the GPC-16 cell model, which acquired an epithelial morphology under recommended growth conditions (**Fig 2B**).

Next, we assessed if the impaired responsiveness of human cells to KDC203 at higher serum levels translates to guinea pig cells. Not only was this the case but the effect was also observed when we expanded analyses to two types of serum, namely horse serum or fetal bovine serum (**Fig 2C**).

Specifically, supplementation of media with 10% concentrations of either serum blocked the KDC203-induced PrP^C^ reduction. When comparing the PI-PLC releasable sub pool of PrP^C^ in 104C1 cells that had been exposed to increasing concentrations of KDC203, a significant reduction of cell surface PrP^C^ levels was readily observable. Interestingly, in contrast to the human cell models, no PrP^C^ was retained in cellular lysates upon PI-PLC treatment, suggesting that in this cell model an insignificant amount of total PrP^C^, below the level of detection by western blotting, is localized within the cells at steady-state levels. (**Fig 2D, lanes 4–6**). A PNGase F-based characterization of the isoform composition of PrP^C^ in 104C1 contributed to the conclusion that the majority of total PrP^C^ in this model exists as N-terminally truncated GPI-anchored cell surface localized C2 fragments (**Fig 2E**).

The above characteristics recommended this cell model as a useful paradigm for analyzing the pharmacodynamics of the KDC203-induced PrP^C^ lowering because the absence of intracellular PrP^C^ would facilitate the western blot-based interpretation of results. To conduct this experiment, we exposed 104C1 cells to varying concentrations of KDC203 for up to 7 days, followed by withdrawal of KDC203 for up to 3 days (**Fig 2F**). Western blot analyses of total cell lysates of cells harvested at various intervals documented a KDC203 time-dependent lowering of steady-state PrP^C^ levels in this model, with the most pronounced reduction seen in cells that were treated for 7 days at 200 nM KDC203 levels (**Fig 2F, lane 11**). Intriguingly, when the treatment was withdrawn, levels of PrP^C^ in previously KDC203 treated cell cultures overshot those observed in mock-treated control cultures (**Fig 2F, compare lanes 14 and 15 with lane 13**). Moreover, the degree to which PrP^C^ levels overshot correlated with the concentration of KDC203 that the respective cells had previously been exposed to, i.e., PrP^C^ levels were about twice as high in cells that had been treated at 400 nM KDC203 levels than in cells that had been exposed to 200 nM KDC203. By the third day of KDC203 withdrawal, PrP^C^ levels normalized in cells that had or had not been treated with KDC203.

Taken together, these experiments suggested that the KDC203 lowering capacity translates to other species than humans, and that cells subjected to KDC203 treatment may activate a compensatory biology that aims to replenish PrP^C^ cell surface levels, causing excessive PrP^C^ levels when the PrP^C^ lowering treatment is withdrawn.

### KDC203 lowers PrP^C^ levels in primary cultures of guinea pig neurons, astrocytes, or cardiomyocytes

Next, we explored the PrP^C^ lowering capacity of KDC203 in primary cells that we extracted from embryonic guinea pig brain and heart tissue and expanded in cell culture. Initially, we assessed the KDC203 lowering capacity in primary neural cultures that comprised cells of astrocytic and neuronal lineage at varying degrees of differentiation.

**Fig 3:**
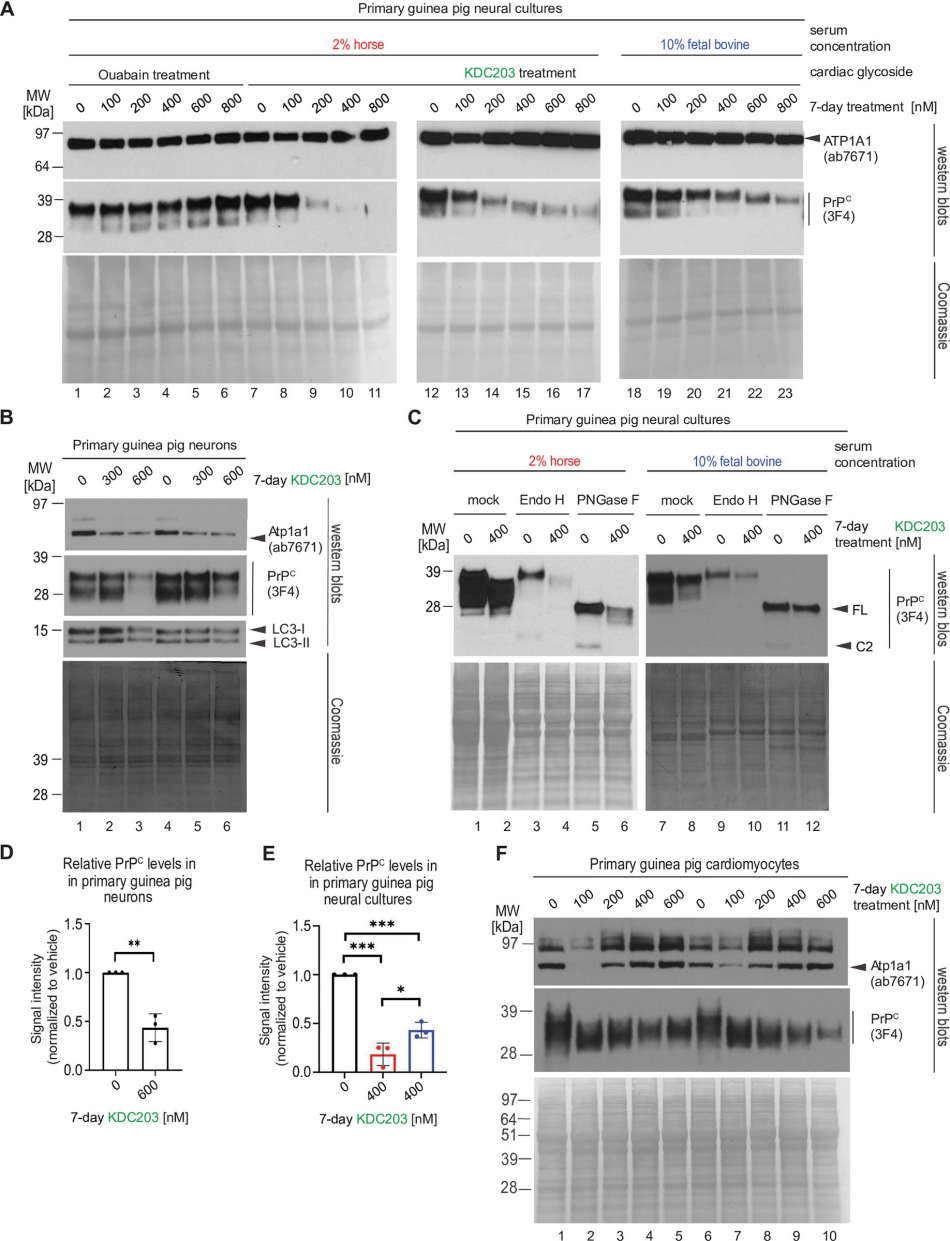
KDC203 lowers PrP^C^ levels in primary cultures of guinea pig neurons, astrocytes, or cardiomyocytes. (A) KDC203, but not Ouabain, lowers steady-state PrP^C^ levels in primary guinea pig neural cultures, with the PrP^C^ lowering potency enhanced in media containing 2% horse serum than 10% fetal bovine serum. (B) Primary neurons respond to KDC203 with the lowering of steady-state Atp1a1 and PrP^C^ protein levels. LC3-directed western blotting does not reveal the molecular autophagy signature even when cells were exposed to 600 nM KCD203 levels. (C) 7-day KDC203 treatment of primary guinea pig astrocytes led to a reduction in steady-state PrP^C^ levels and a downward shift in its apparent molecular weight that was more pronounced in 2% horse serum than 10% fetal bovine serum. The subsequent digestion with endoglycosidase H (Endo H) or PNGase F failed to reveal a shift in the apparent MW of PrP^C^ in response to Endo H yet documented a profound shift upon PNGase F digestion, consistent with KDC203 at 400 nM having had no impact on the ability of PrP^C^ to reach the cell surface. Note that this analysis did not consider relative signal intensities prior to and after digestion (likely caused by a reduced propensity of PrP^C^ to transfer to the western blot transfer upon removal of its N-glycans). (D) Densitometric western blot quantitation of steady-state PrP^C^ levels in primary guinea pig neurons following 7-day KDC203 treatment, including the results shown in Panel B. (E) Densitometric western blot quantitation of steady-state PrP^C^ levels in primary guinea pig neural cell cultures that were for 7 days exposed to vehicle or 400 nM KDC203 in the presence of 2% horse serum (red bar) or 10% fetal bovine serum (blue bar), including the results shown in Panels A and C. (F) 7-day KDC203 treatment of primary guinea pig cardiomyocytes led to a bimodal shift in the steady-state levels of Atp1a1 that was most pronounced at 100 nM treatment concentrations. In contrast, the signal intensities of PrP^C^ western blot bands declined in a KDC203 concentration-dependent manner that was accompanied by an increase in their mobility in samples derived from KDC203-treated cardiomyocytes. Two biological replicates were included for each KDC203 concentration to document the reproducibility of these complex changes to PrP^C^ and Atp1a1 signals in this cell model.

To supplement the cells with sufficient nutrients we evaluated their response to increasing KDC203 or Ouabain concentrations in 2% horse serum (**Fig 3A**). Western blot analyses revealed these cultures to be unresponsive in their PrP^C^ levels to Ouabain but exhibiting dose-dependent reductions in their PrP^C^ levels when exposed to KDC203. As seen in other paradigms, increasing serum levels to 10% fetal bovine reduced the potency of PrP^C^ reduction (**Fig 3A,** compare lanes 14 and 20).

For treating human prion diseases, a PrP^C^ lowering strategy would need to also be effective on neurons. Concerned that primary neural cultures grown in the presence of high serum concentrations tend to be dominated by astrocytes, we next subjected these neural cultures to growth conditions that selectively kill cells of astrocytic lineage, then treated the remaining neuronally enriched cultures with KDC203 (**Fig 3B**). Western blot analyses documented that Atp1a1 levels were already diminished at 300 nM KDC203 levels and that addition to the media of 600 nM KDC203 also diminished steady-state levels of PrP^C^. Probing for the microtubule-associated protein light chain 3B in its lipid modified form (LC3-II) did not point toward enhanced autophagy in KDC203 treated cells.

Undertaking 400 nM KDC203 treatments with primary neural cultures grown in media containing 2% horse serum, caused total PrP^C^ bands to migrate faster than the corresponding bands in mock treated cells (**Fig 3C**). PNGase F digestions revealed the isoform identify of the respective bands as predominantly full length. To understand if the slower PrP^C^ migration was a result of KDC203 influencing the post-translational maturation of PrP^C^ during its passage through the secretory pathway, we subjected cell lysates to digestion with endoglycosidase H (Endo H), then compared whether the slower migrating bands were subject to an additional increase in mobility, which would be expected if the high mannose N-glycosylated PrP^C^ molecules had been prevented by KDC203 from maturing in the Golgi into complex glycan structures (**Fig 3C**, compare lanes 2 and 4, in the higher exposed film shown at the bottom). No such additional shift was observed, pointing toward a preferential degradation of fully glycosylated PrP^C^, rather than an immature maturation, as the more plausible explanation for the slower migration of bands in KDC203 treated cultures. As seen in other paradigms, changing to a 10% serum containing medium interfered with the KDC203-dependent mobility shift and the reduction of PrP^C^ levels also in this model. Quantifications of the PrP^C^ lowering effect sizes of KDC203 in guinea pig neural cultures and primary neurons revealed them to be significant based on densitometric analyses of western blot bands of three biological replicates (**Fig 3D and 3E**).

In preparation of *in vivo* KDC203 treatments of guinea pigs, we next explored if a PrP^C^ lowering capacity of KDC203 could perhaps also be observed in cells outside the brain. To this end, we treated primary cardiomyocytes that we had harvested from heart tissue and expanded them in cell culture followed by exposure to various concentrations of KDC203. This experiment revealed a complex bimodal response of Atp1a1 levels to increasing KDC203 concentrations and revealed increases in the relative mobility of PrP^C^, with 100 nM KDC203 concentrations sufficient to increase mobility significantly, and further increases in KDC203 concentrations leading to lowering of steady-state PrP^C^ signal intensities (**Fig 3F**).

### Subcutaneous *in vivo* administration of KDC203 to guinea pigs lowered steady-state PrP^C^ levels in heart but not in brain tissue

**Fig 4:**
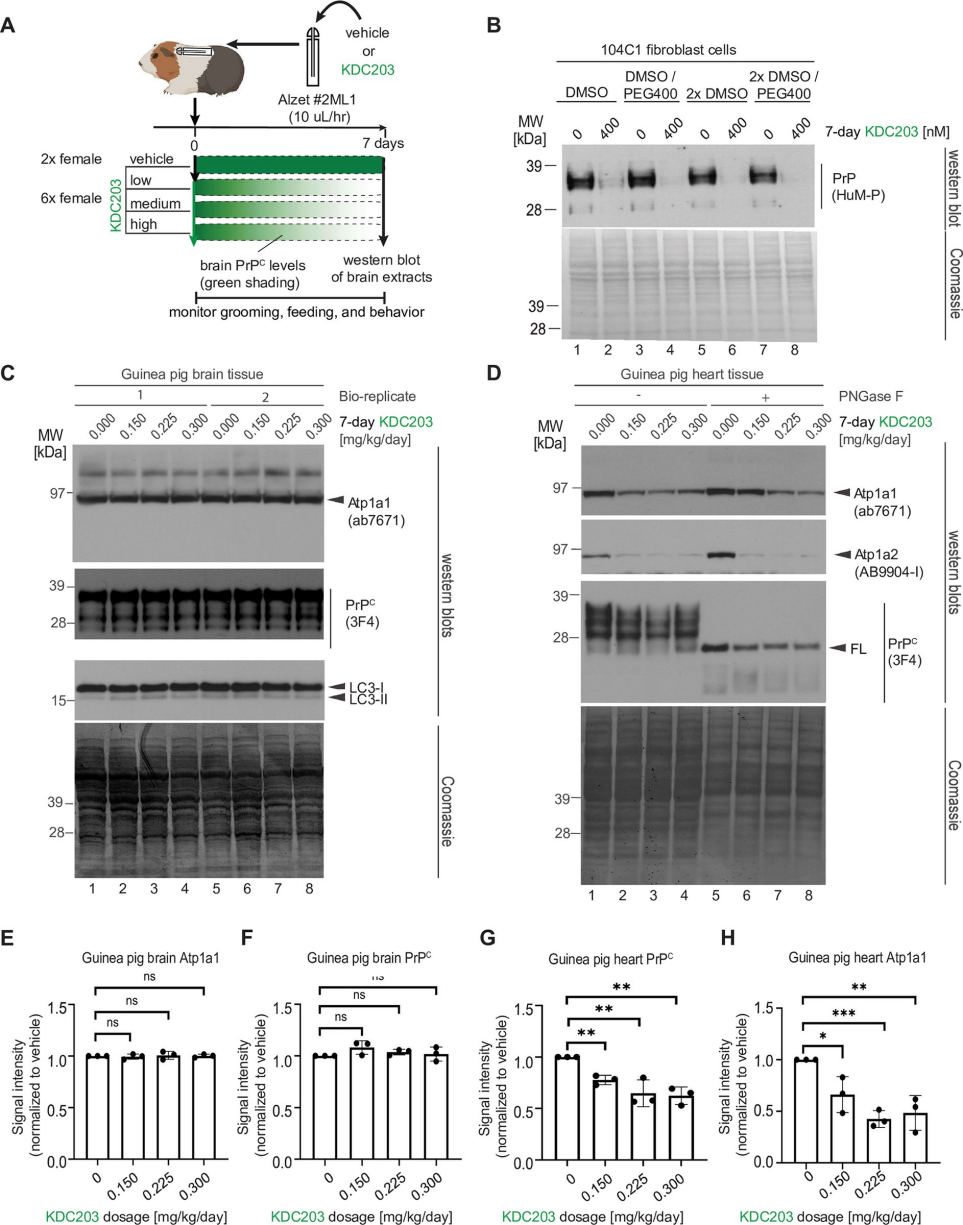
Subcutaneous administration of KDC203 to guinea pigs lowered steady-state PrP^C^ levels in the heart but not in brain tissue. (A) Cartoon depicting design of guinea pig KDC203 treatment study. (B) Analysis of the impact of KDC203 formulation on its ability to lower steady-state PrP^C^ levels in 104C1 guinea pig cells. (C) 7-day subcutaneous administration of KDC203 to guinea pigs did not change steady-state guinea pig brain Atp1a1 or PrP^C^ levels or cause a molecular LC3 signature that indicated autophagy in brain tissue. (D) 7-day subcutaneous administration of KDC203 to guinea pigs lowered steady-state Atp1a1, Atp1a2 and PrP^C^ protein levels in heart tissue. PNGase F digestion of extract proteins revealed that total PrP^C^ levels in this tissue were predominantly comprised of full-length PrP^C^. (E-H) Subcutaneous 7-day administration of KDC203 in guinea pigs revealed no significant effect of KDC203 on the brain but a significant dose-dependent effect on the heart. Densitometric quantitation of steady-state brain and heart Atp1a1 levels (E, G) and PrP^C^ levels (F, H).

Next, we undertook a small dose-finding study to reveal if systemically administered KDC203 can be tolerated and can lower PrP^C^ levels in guinea pig tissues of interest after subcutaneous *in vivo* delivery. The choice to base this experiment on subcutaneous, as opposed to intracerebral injections, reflected an intent to mimic systemic treatment routes, which have been in the clinic for humans and other mammals for many years. To minimize peak-trough effects that tend to manifest after bolus injections, we surgically implanted osmotic pumps (Alzet) subcutaneously so that they would deliver four doses of KDC203 (0.00 mg/kg/day, 0.15 mg/kg/day, 0.225 mg/kg/day, and 0.30 mg/kg/day) continuously for a duration of seven days. Each of the four cohorts comprised two female guinea pigs (**Fig 4A**). We had already reported that KDC203 remains stable when incubated at 37% for two weeks (see Supplementary Materials in [22]). Here, we validated that its PrP^C^ lowering potency in our cell-based assay is not influenced by the presence of a 1:1 ratio of DMSO and PEG400 (v/v), which we selected as the base formulation for solubilizing and storing KDC203 in the Alzet pump during the experiment (**Fig 4B**).

Following the 7-day KDC203 treatment, guinea pigs were euthanized, and downstream analyses focused on brain, and heart tissues, based on their well-established prominent levels of NKA expression. Western blot analyses of brain homogenates from the eight guinea pigs revealed no significant difference in the steady-state levels of Atp1a1, PrP^C^ or LC3 isoforms I and II (**Fig 4C**). In contrast, analyses of guinea pig heart tissue homogenates revealed lower steady-state levels of the NKA α subunits Atp1a1 and Atp1a2 (note that Atp1a3 is not prominently expressed in the heart) in KDC203-treated guinea pigs. Steady-state PrP^C^ levels were lowered to a lesser, significant degree in these heart homogenates (**Fig 4D**). No indication of systemic KDC203 administration having triggered autophagy was observed. To focus the complex western blot signals that can be assigned to PrP^C^ due to its post-translational N-glycosylation, we reanalyzed the heart homogenate samples following PNGase F digestion. This step revealed heart PrP^C^ to be composed primarily of the full-length isoform, which appears to undergo limited endoproteolysis. Importantly, this analysis corroborated the observation that steady-state PrP^C^ levels were lower in the hearts of KDC203-treated guinea pigs. The same trend of slightly diminishing PrP^C^ signals in KDC203-treated animals was also observed in lung tissue (**S1 Fig**).

Taken together, the results from this *in vivo* dose finding study suggested that the subcutaneous delivery of KDC203 led to insufficient KDC203 brain levels for the PrP^C^ lowering effect to manifest there (**Fig 4E and 4F**), yet it also validated that systemic KDC203 administration can cause a significant reduction of its NKA α subunit targets in the easier accessible tissues (**Fig 4G and 4H**).

### KDC203 lowers PrP^C^ levels in cultured guinea pig cortical and cerebellar brain slices

**Fig 5:**
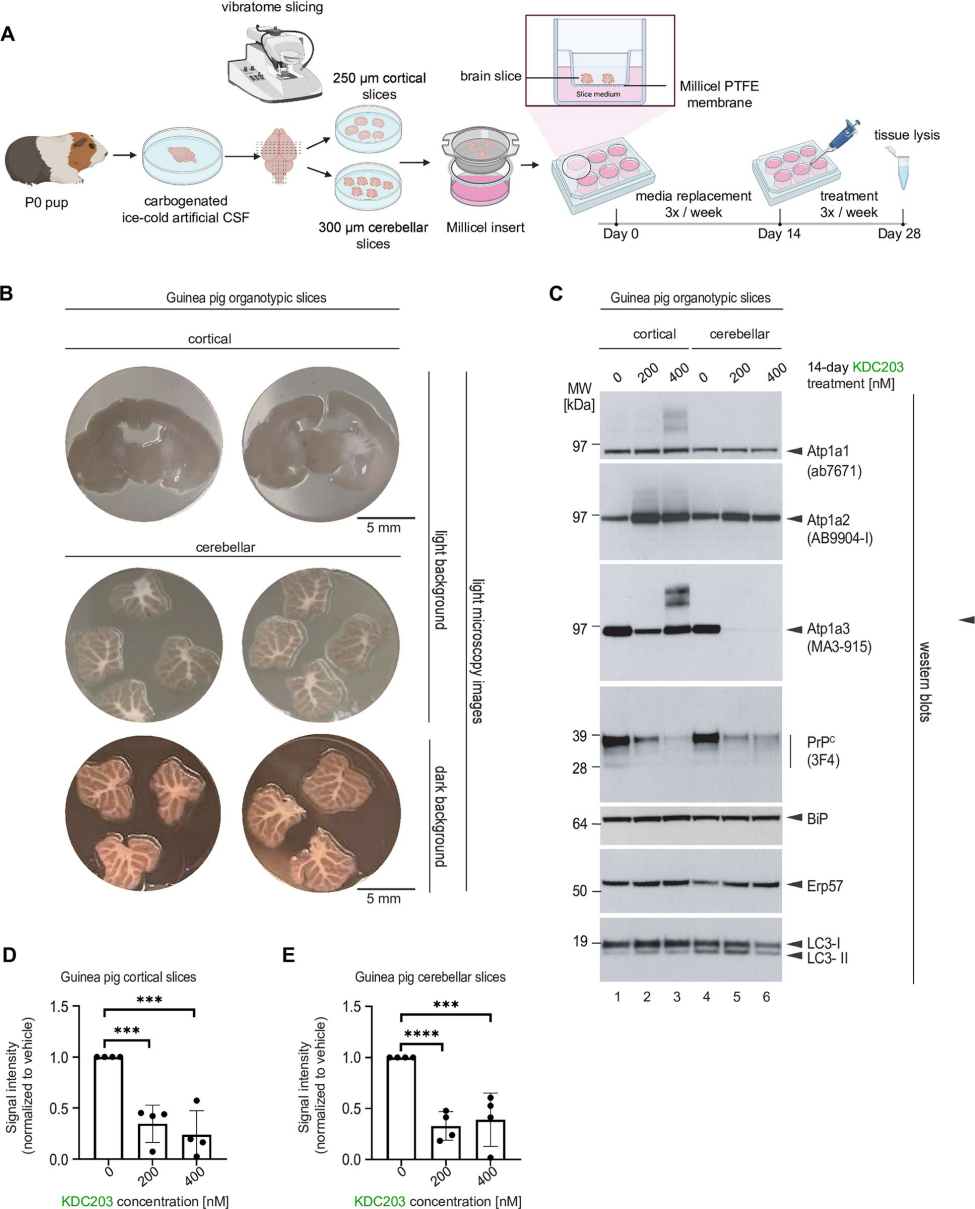
KDC203 lowers PrP^C^ levels in cultured guinea pig cortical and cerebellar brain slices. (A) Cartoon depicting the workflow for generating cortical and cerebellar guinea pig organotypic brain slices. (B) Microscopy images of cortical and cerebellar guinea pig organotypic brain slices. (C) Western blot analyses of proteins-of-interest following 14-day treatment without or with 200 nM or 400 nM KDC203 in the brain-in-a-dish culture medium. Note that KDC203 exhibited inconsistent effects on the three NKA α subunits, with steady-state Atp1a1 levels being the least affected, Atp1a2 levels elevated in both cortical and cerebellar slices exposed to KDC203, and Atp1a3 levels being differently affected in cortical and cerebellar slices by the presence of KDC203. In contrast, steady-state PrP levels were consistently lower in a KDC203 concentration-dependent manner in both cortical and cerebellar organotypic brain slices. Finally, markers of endoplasmic reticulum stress, BiP and Erp57, or autophagy, LC3, were not significantly altered in the two types of brain slices with or without KDC203 exposure. (D, E) Quantitation of steady-state PrP^C^ levels in vehicle-treated or KDC203-treated cortical (D) or cerebellar (E) brain slices. Significance thresholds indicated in the graphs are based on densitometric analyses of western blot signal intensities from four biological replicates for each treatment group.

Next, we explored if KDC203 can lower PrP^C^ levels also in brain cells that are embedded in their natural three-dimensional brain architecture. To implement this plan, without having to achieve >100 nM levels of penetrance of the blood-brain barrier, we turned to organotypic cortical and cerebellar brain slices grown *ex vivo*. To this end, we adopted a protocol for the culture of mouse organotypic brain slices to working with guinea pig brains (**Fig 5A**). More specifically, dissected brains of P0 guinea pig pups were temporarily preserved in ice-cold artificial CSF, then sliced with a vibratome, and maintained on Millicel inserts for two weeks. Next, we exposed cortical or cerebellar guinea pig slices for durations of 14 days to 200 nM or 400 nM KDC203 in the brain slice culture medium. During this time, the brain slices thinned and developed satellite colonies on their edges that are characteristic for healthy organotypic cultures (**Fig 5B**). Mammalian brains, in contrast to several cell models, are known to express three NKA α subunits that are encoded by Atp1a1, Atp1a2, and Atp1a3 genes with distinct pharmacological profiles for CG binding. To capture this redundancy, we probed western blots with antibodies that were specific for one of these three subunits. This analysis revealed that the tissue indeed had responded to KDC203 with response profiles that were specific for each NKA α subunit: Whereas steady-state Atp1a1 levels remained unchanged in the presence of KDC203, Atp1a2 levels increased, and Atp1a3 showed a bimodal KDC203 response in its steady-state levels in cortical slices and was mostly depleted in the presence of KDC203 in cerebellar slices (**Fig 5C**).

In contrast to this relatively complex response of NKA α subunits, KDC203 lowered steady-state PrP^C^ levels in a dose dependent manner in both cortical and cerebellar guinea pig slices (**Fig 5D and 5E**). To investigate if the reduced PrP^C^ levels merely reflected slowed biosynthesis of many proteins in the presence of KDC203, we also analyzed the same samples with antibodies against two endoplasmic reticulum (ER) marker proteins, i.e., BiP and Erp57, whose own levels can be responsive to defects in protein homeostasis (**Fig 5C**, see also **S2 Fig** for uncropped images). Although the levels of Erp57 and BiP seem to subtly increase in the presence of KDC203, these level changes did not reach significance for these two proteins. Finally, we assessed if the brain slices exhibited signs of restricted access to nutrients, which often causes starvation-induced autophagy [26,27]. An analysis of LC3-II levels revealed them to be unchanged, consistent with the interpretation that autophagy was not a major factor. Taken together, these results suggested that the brain architecture does not resist the PrP^C^ lowering capacity of KDC203 so long as sufficient levels of this CG can be delivered to the brain.

## Discussion

In this study, we set out to assess the merits of a PrP^C^ lowering strategy for the treatment of prion diseases based on a small molecule. Specifically, we focused on KDC203, a semi-synthetic derivative of the natural cardiac glycoside oleandrin. We observed KDC203 to lower cell surface PrP^C^ levels in a dose-dependent manner in human and guinea pig cells. In a subset of cell types, this PrP^C^ lowering capacity of KDC203 became apparent only upon release of the cell surface pool of PrP^C^ by PI-PLC. An attempt to lower guinea pig brain PrP^C^ levels by continuous subcutaneous KDC203 administration failed. Encouragingly, however, heart and lung PrP^C^ levels were lowered by KDC203 in the same guinea pigs, and brain PrP^C^ levels could be lowered by KDC203 *ex vivo* in organotypic brain slices.

Our work brought to the fore three intricacies of PrP^C^ biology that may come to bear also on a subset of alternative PrP^C^ lowering treatment modalities.

1. The side-by-side comparison of human cell lines revealed that exposure to KDC203 caused most cell types (e.g., T98G cells) to lower total steady-state PrP^C^ levels but others (e.g., LN-226 cells) to increase them. Although not thoroughly investigated here, based on our prior data [21] and results obtained with Endo H in this study (**Fig 3**), we attribute this divergence in outcomes primarily to differences in the rate of degradation of PrP^C^ across cell types upon its CG-induced cellular internalization. Critically, even in cell lines that responded to KDC203 with an increase in total PrP^C^ levels, the PI-PLC releasable pool of cell surface PrP^C^ was still reduced. All available data suggest that lowering this pool of cell surface PrP^C^ is needed to reduce interactions of the prion disease-associated conformers of the prion protein with the cell surface, thereby slowing the spread of the disease [28–30]. The cell surface pool of PrP^C^ is also widely regarded as critical for conferring toxicity in prion diseases [31,32], and perhaps even in other dementias, in which PrP^C^ has been proposed to represent a prominent receptor for oligomeric assemblies of disease-associated proteins, including oligomeric forms of Aβ [33–39]. It remains unresolved at this time if the accumulation of PrP^C^ in cells that exhibit lower rates of degradation has the propensity to become problematic if CG treatments were to be administered for extended periods of time. This will need to be closely looked at in a future *in vivo* study.
2. In several *in vitro* paradigms, the capacity of KDC203 to lower cell surface PrP^C^ levels was influenced by the serum concentration of the growth medium. Our results suggest that two phenomena contributed to this outcome to different degrees in the various paradigms we assessed. In a subset of models, cells grown in richer medium were observed to express higher PrP^C^ steady-state levels at baseline. This was the case in LN-229 cells (**Fig 1D**) and in 104C1 guinea pig cells (**Fig 2C**), thereby raising the bar for any PrP^C^ lowering strategy. In other instances, including T98G cells, this explanation is not well supported by our data, which revealed PrP^C^ bands of similar intensity in media with different serum concentrations prior to any KDC203 treatment (**Fig 1C**). Here, the reduced PrP^C^ lowering capacity of KDC203 in serum rich media is easier reconciled with the interpretation that a larger fraction of KDC203 may be rendered ineffective by components in the serum, e.g., through sequestration by serum proteins or lipids, thereby precluding its binding to NKAs.
3. Work with guinea pig cells established that withdrawal of KDC203 after one week of daily administration caused PrP^C^ levels to temporarily overshoot (**Fig 2F**), thereby revealing a compensatory biology that was masked during the treatment period. This kind of feedback biology can be encountered at every turn in biological systems, and is consistent with a prior report of a PrP lowering compound causing a dose-dependent increase in PrP expression in a mouse cell paradigm [40]. Its existence may be inconsequential for gene therapy approaches designed to block the transcription of PrP^C^ (e.g., ZFPs) but should be considered in other PrP^C^ lowering modalities, as it might accentuate undesired peak-trough PrP^C^ fluctuations when treatments are administered intermittently.

A hopeful result from this study is the observation that KDC203 lowered cell surface PrP^C^ levels in all *in vitro* and *ex vivo* paradigms we assessed, including organotypic cortical and cerebellar slices. To the best of our knowledge, this report also is the first to establish that a systemically administered small molecule of a clinically well-established compound class can lower PrP^C^ levels *in vivo*.

The work we presented has limitations: Notably, it did not achieve one of its main goals, namely, to identify a suitable *in vivo* rodent model for testing if systemically administered KDC203 can prolong survival in prion diseases. There are several possible explanations for why KDC203 did reduce PrP^C^ levels in guinea pig hearts and lungs but not in the brain. We had previously shown that KDC203 can reach approximately 30 nM brain levels upon subcutaneous injection into mice [22]. Data in this work corroborated that KDC203 concentrations below 30 nM are sufficient for lowering PrP^C^ levels in human cells, but concentrations exceeding 100 nM were required for lowering PrP^C^ levels in guinea pigs. If the ability of KDC203 to penetrate the blood-brain-barrier and distribute within the brain is similar amongst rodents, including mice and guinea pigs, these results suggest that the most parsimonious interpretation for not having reached efficacy in guinea pig brains is a reality whereby KDC203 brain levels in this organism probably remained below 100 nM. This conclusion is corroborated by our separate observation that guinea pig *ex vivo* organotypic brain slices exhibited dose-dependent reductions in the steady-state levels of PrP^C^ once exposed to 200 nM or 400 nM KDC203 levels, suggesting that lowering PrP^C^ levels in the brain using a CG-based small molecule may be possible so long as a delivery route is used that manifests in effective unbound KDC203 brain levels exceeding the concentration required for target engagement. Encouragingly, the levels of KDC203 required to lower human cell surface PrP^C^ levels were confirmed in this study to fall below those we had observed to penetrate the blood brain barrier [22], keeping the door open for further exploration in a more suitable pre-clinical animal.

## Supporting information

S1 Fig Subcutaneous administration of KDC203 to guinea pigs lowered steady-state PrPC levels in lung tissue in a dose dependent manner. (A) PrP^C^ levels in guinea pig lung tissue protein extracts, without and with PNGase F digestion. (B) Quantitation of steady-state lung PrP^C^ levels in response to subcutaneous 7-day administration of KDC203 in guinea pigs revealed a significant KDC203 dose-dependent effect on the lung.
(PDF)

S2 Fig Raw western blot and Coomassie images. (PDF)
