# Supplementary figures and images for: Update on a brain-penetrant cardiac glycoside that can lower cellular prion protein levels in human and guinea pig paradigms
Source: PLoS One. 2024 Sep 24;19(9):e0308821. doi: 10.1371/journal.pone.0308821 (PMC11421771; doi:10.1371/journal.pone.0308821)

S1 Fig

A

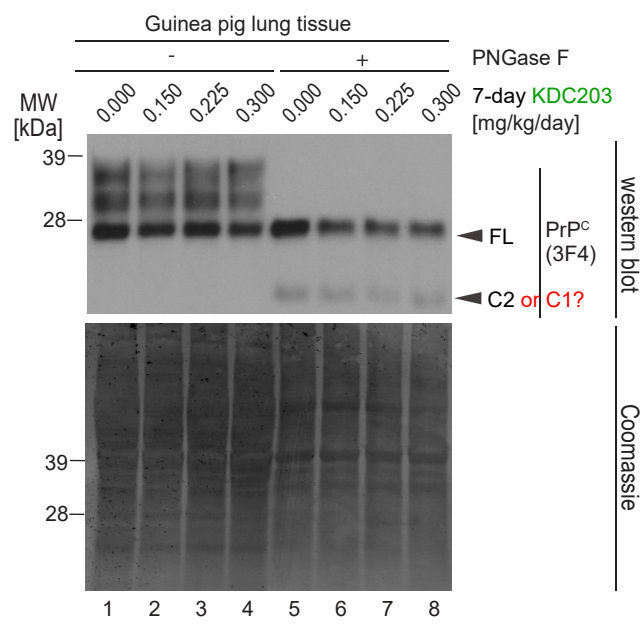

B

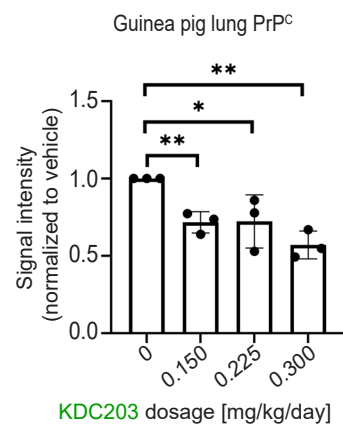

Supplement: S1 Fig — (A) PrPC levels in guinea pig lung tissue protein extracts, without and with PNGase F digestion. (B) Quantitation of steady-state lung PrPC levels in response to subcutaneous 7-day administration of KDC203 in guinea pigs revealed a significant KDC203 dose-dependent effect on the lung. (PDF) [file pone.0308821.s001.pdf]

Figure 1

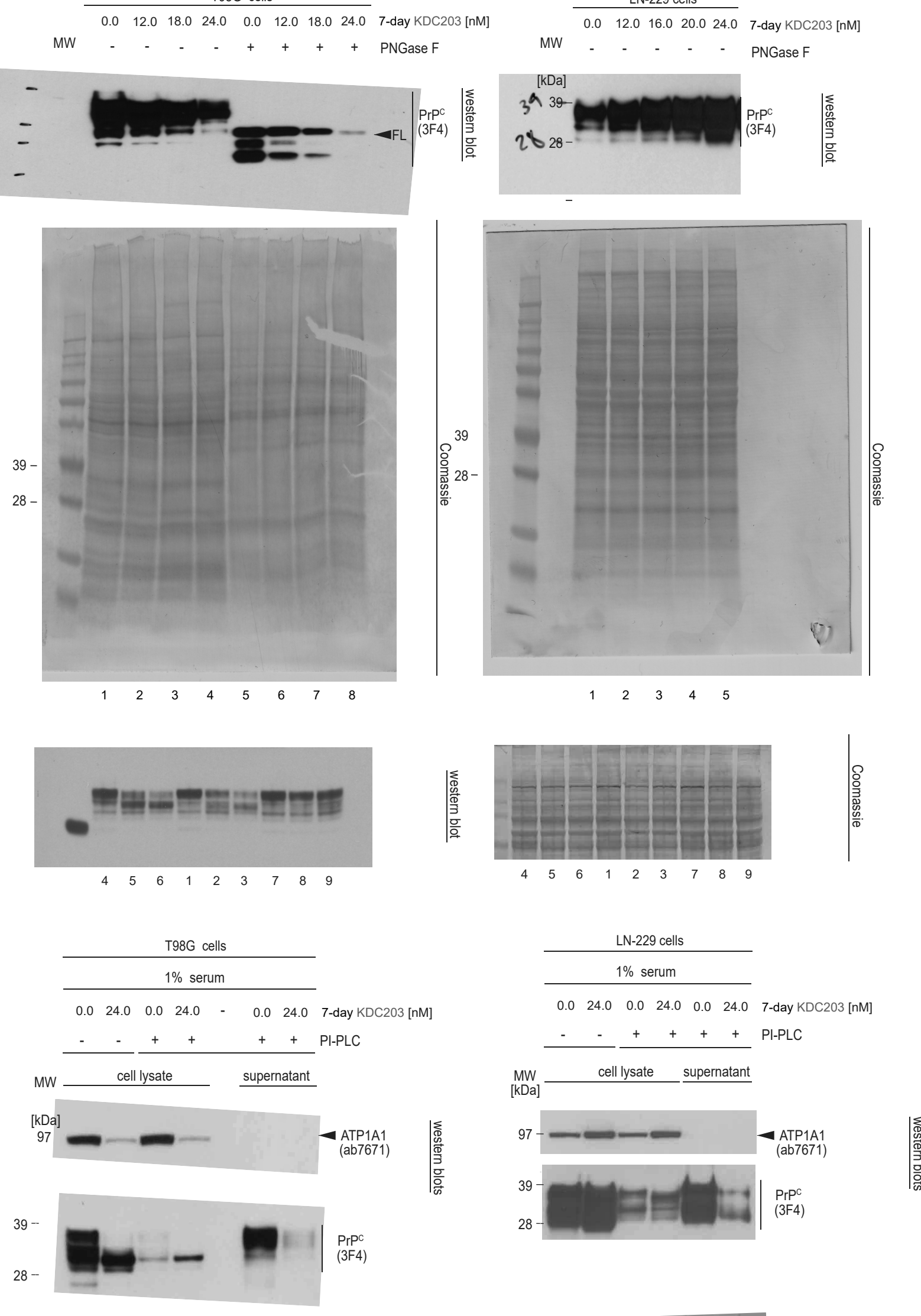

Figure 2

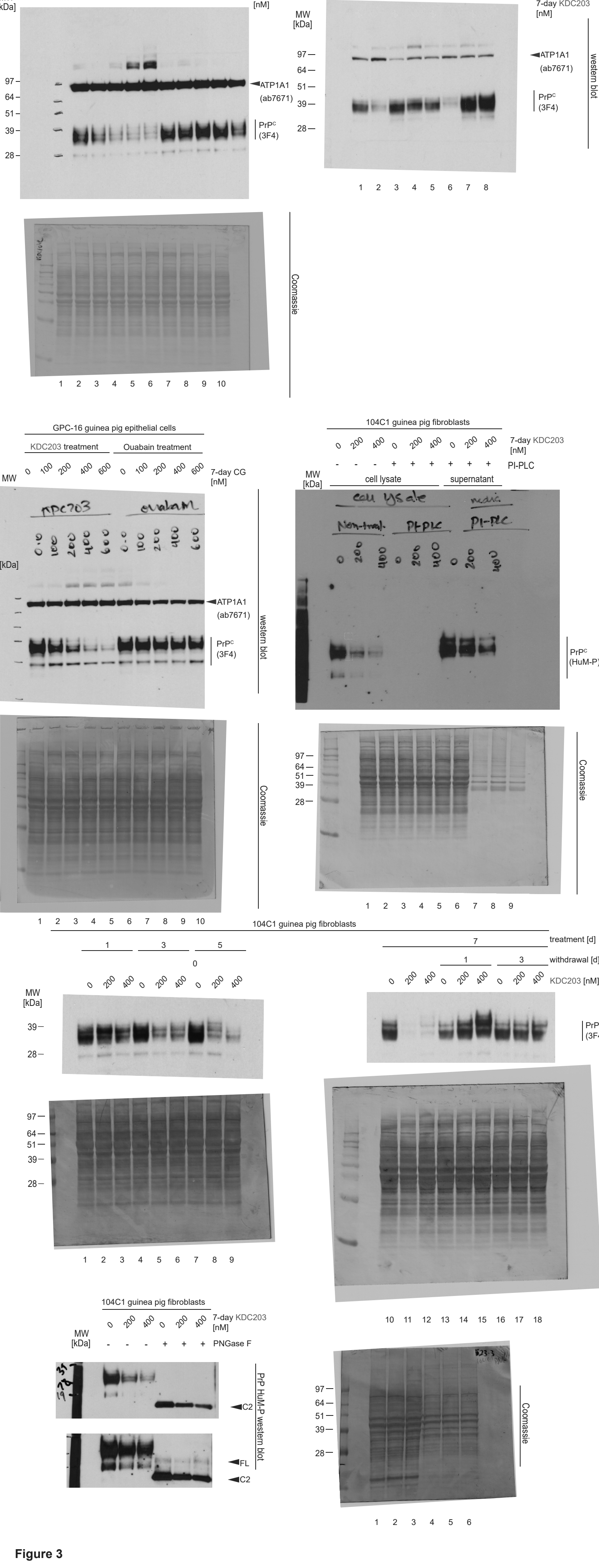

Figure 3

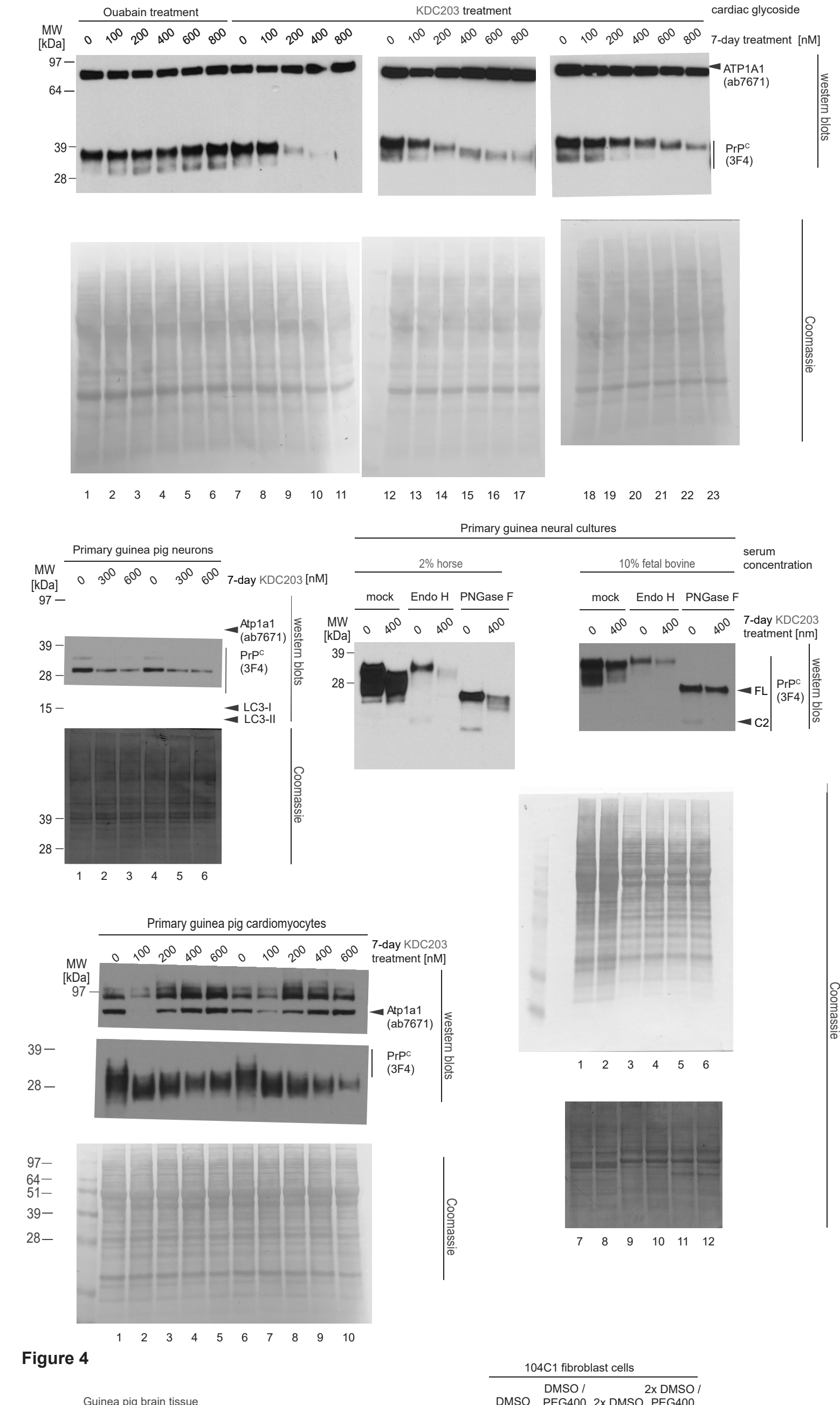

Figure 4

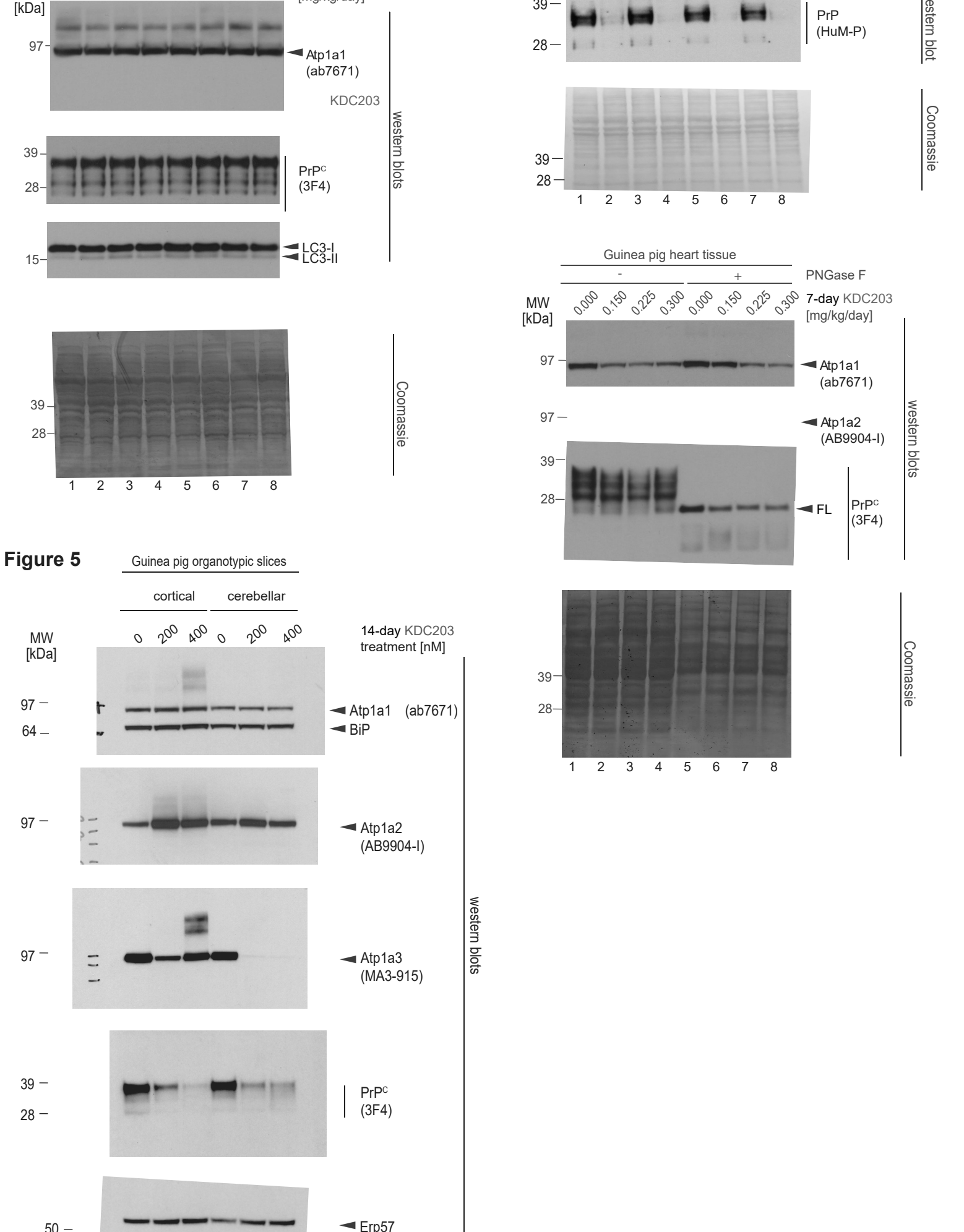

Figure 5

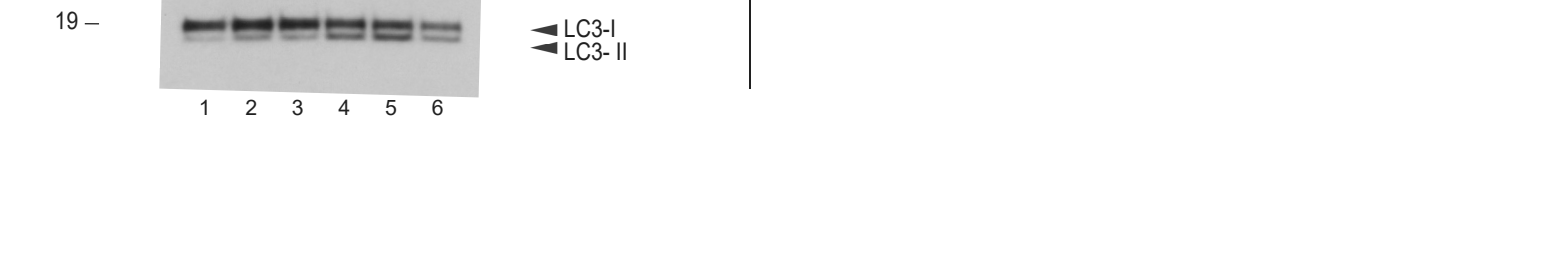

Supplement: S2 Fig — (PDF) [file pone.0308821.s002.pdf]
